# Oncogenes and RNA splicing of human tumor viruses

**DOI:** 10.1038/emi.2014.62

**Published:** 2014-09-03

**Authors:** Masahiko Ajiro, Zhi-Ming Zheng

**Affiliations:** Tumor Virus RNA Biology Section, Gene Regulation and Chromosome Biology Laboratory, Center for Cancer Research, National Cancer Institute, National Institutes of Health, Frederick, MD 21702, USA

**Keywords:** EBV, HBV, HCV, HPV, HTLV-1, KSHV, MCV, RNA splicing

## Abstract

Approximately 10.8% of human cancers are associated with infection by an oncogenic virus. These viruses include human papillomavirus (HPV), Epstein–Barr virus (EBV), Merkel cell polyomavirus (MCV), human T-cell leukemia virus 1 (HTLV-1), Kaposi's sarcoma-associated herpesvirus (KSHV), hepatitis C virus (HCV) and hepatitis B virus (HBV). These oncogenic viruses, with the exception of HCV, require the host RNA splicing machinery in order to exercise their oncogenic activities, a strategy that allows the viruses to efficiently export and stabilize viral RNA and to produce spliced RNA isoforms from a bicistronic or polycistronic RNA transcript for efficient protein translation. Infection with a tumor virus affects the expression of host genes, including host RNA splicing factors, which play a key role in regulating viral RNA splicing of oncogene transcripts. A current prospective focus is to explore how alternative RNA splicing and the expression of viral oncogenes take place in a cell- or tissue-specific manner in virus-induced human carcinogenesis.

## INTRODUCTION

Infection with human oncogenic viruses is the cause of ∼10.8% of human cancers worldwide.^1^ The first oncogenic virus to be identified, avian Rous sarcoma virus, was discovered by Peyton Rous in 1911.^2^ Decades later, a series of other oncogenic viruses were also discovered, including cottontail rabbit papillomavirus,^3^ mouse mammary tumor virus,^4^ adenovirus,^5,6^ and simian virus 40 (SV40).^7^ The theory of virus-mediated oncogenesis was finally experimentally demonstrated in 1976 by Harold Varmus and Michael Bishop through the identification of *v-src* as the Rous sarcoma virus viral oncogene.^8^ Characterization of viral oncogenes also brought numerous landmarks in understanding of the oncogenic process, such as the discoveries of p53 as an SV40 T-antigen-associated protein^9,10^ and E2F as a mediator of adenovirus E1A function.^11^

Although the oncogenic viruses found in the early studies exhibited oncogenic activity in animal cells, they failed to transform human cells. The theory of viral oncogenesis in humans remained controversial until 1965, when Epstein–Barr virus (EBV) was discovered in Burkitt lymphoma cells (Table 1).^12^ Subsequent demonstrations, including isolation of human T-cell lymphoma virus-1 (HTLV-1) from adult T-cell lymphoma (ATL)^13,14,15^ and the association of high-risk human papillomaviruses (HPVs) with cervical cancers,^16,17,18,19^ paved the way for the concept of human tumor viruses. Discovery of the association of Kaposi's sarcoma-associated herpes virus (KSHV) with Kaposi's sarcoma^20^ and lymphoma,^21^ and of Merkel cell polyomavirus (MCV) with Merkel cell carcinoma (MCC)^22^ underscores the possibility that even more tumor viruses will be discovered by modern technology. These tumor-inducing human viruses encode viral oncogenes or genes with oncogenic activities and utilize cellular machinery for their expression and the transformation of host cells. One of the indispensable steps for viral oncogene expression is RNA splicing, which is essential for almost all tumor viruses to diversify their transcriptomes during virus infection and oncogenesis. This review will highlight the recent advances in understanding of human viral oncogenes and the importance of RNA splicing in their expression.

## HUMAN PAPILLOMAVIRUS

### HPV and oncogenic activity

HPVs are a group of non-enveloped double-stranded DNA viruses that preferentially infect the cells in the basal layer of skin and mucosal tissues, primarily through microtraumas or close contact. More than 180 HPV genotypes have been reported (http://pave.niaid.nih.gov). HPVs associated with cancers are referred to as high-risk HPVs, and those associated with benign anogenital or skin warts are low-risk HPVs.^23,24^ To date, more than 95% of cervical cancers, 50%–90% of other anogenital cancers and 20%–30% of oropharyngeal cancers have been associated with persistent infection and genomic integration of high-risk HPVs.^24,25,26^ Among the common high-risk HPVs (HPV16, 18, 31, 33, 45 and 58), HPV16 is the most prevalent genotype and is responsible for ∼60% of cervical cancer cases worldwide.^18,26^ The HPV16 viral genome encodes eight open reading frames (ORFs). Six (E1, E2, E4, E5, E6 and E7) are encoded from the early region and two (L1 and L2) are encoded from the late region of the virus genome. E6 and E7 are responsible for the oncogenic activities of high-risk HPVs.

**Figure 1:**
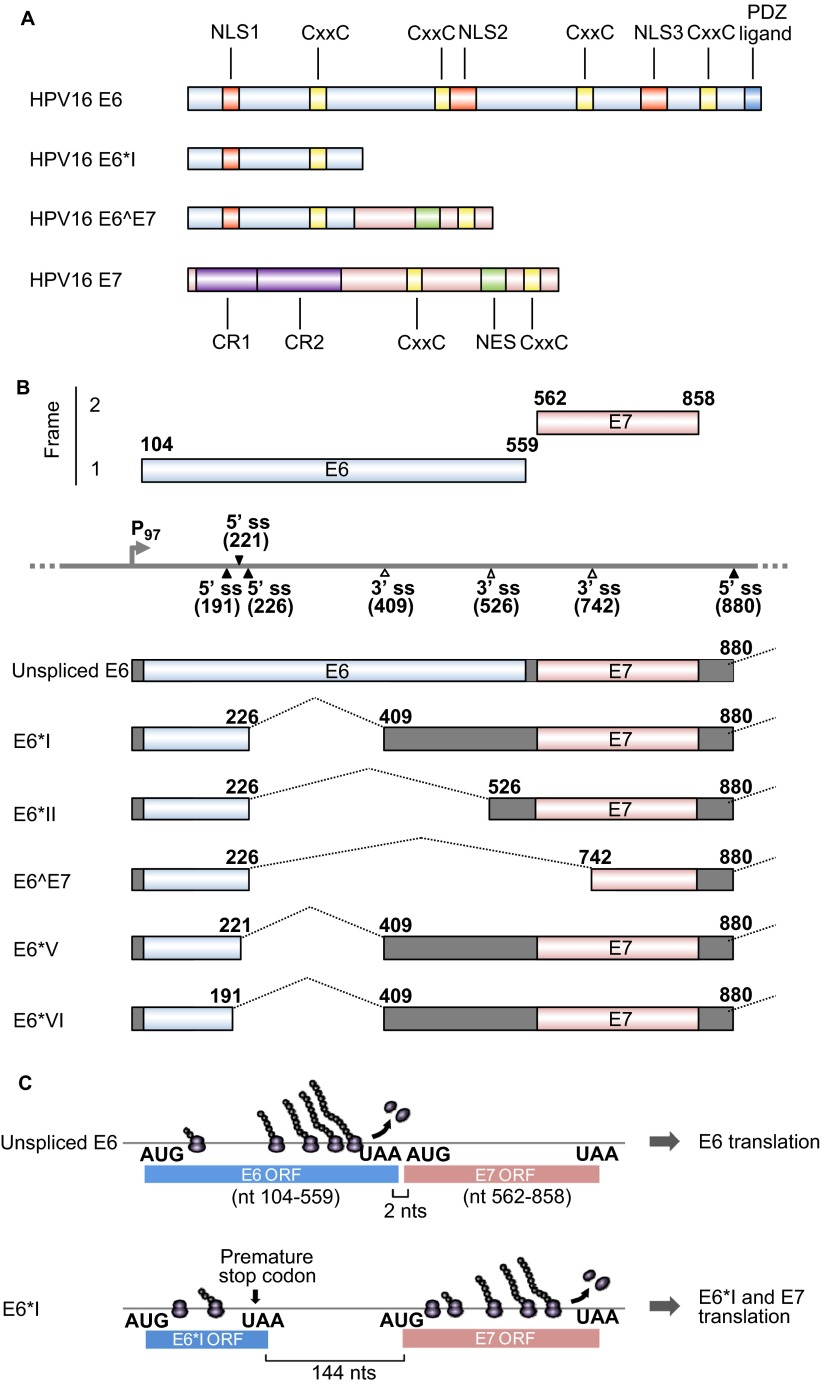
HPV16 E6 and E7 and alternative RNA splicing. (**A**) Major functional domains and motifs of the HPV16 E6, E6*I, E6̂E7 and E7 proteins. HPV16 E6̂E7 has the N-terminal half of E6 and the C-terminal half of E7. (**B**) Alternative RNA splicing products of HPV16 E6E7 pre-mRNA. Alternative RNA splicing (dashed lines) takes place from three 5′ ss at nt 191, 221 and 226 to three alternative 3′ ss at nt 409, 526 and 742.^27^ The majority of RNA splicing occurs from the nt 226 5′ ss to the nt 409 3′ ss to produce E6*I, which is responsible for E7 translation.^28^ E6*III derived from the nt (226 5′ ss to the nt 3358 3′ ss and E6*IV derived from the nt 226 5′ ss to the nt 2709 3' ss^29^ are not included in this diagram. (**C**) Illustration of a ribosomal scanning model in HPV16 E6 and E7 translation, which is regulated by RNA splicing. Full-length E6 is translated from the unspliced E6 mRNA (upper diagram). E7 (nt 562–858) is translated from spliced E6*I mRNA in which a premature stop codon (UAA) is introduced by RNA splicing to form the E6*I ORF (lower diagram), which enlarges the space between the two ORFs. This enables a scanning ribosome to terminate translation of E6*I and reinitiate translation of E7. Nucleotide positions are numbered according to the HPV16 reference genome (PaVE, http://pave.niaid.nih.gov).

The ∼18 kDa HPV16 E6 oncoprotein consists of ∼150 amino acid (aa) residues with four CxxC zinc-binding motifs (Figure 1A), which form two hypothetical zinc fingers. The C-terminus of HPV16 E6 has a PSD-95/disks large/zonula occludins (PDZ)-binding domain and interacts with several PDZ proteins, such as SAP97/hDlg^30,31^ and MAGI-1.^32,33^ HPV16 E6 is targeted to the nucleus through its three nuclear localization signals,^34^ and its oncogenic activity depends mainly on E6-mediated p53 degradation. E6 from high-risk HPVs, but not from low-risk HPVs, interacts with E3 ubiquitin ligase E6-associated protein (E6AP) and promotes the target recognition of E6AP, which leads to ubiquitination of p53.^35^ HPV16 E6 appears to be more potent than HPV18 E6 for p53 degradation.^35^ E6AP binds to E6 through its acidic LxxLL motif and stabilizes the E6 protein.^36,37,38^ The N-terminal region of HPV16 E6 is responsible for E6 homodimerization, which is necessary for the p53-targeting activity of HPV16 E6.^39^ The F47 residue within the N-terminal hydrophobic interface of HPV16 E6 is essential, and mutation of this residue results in loss of the p53 degradation activity of E6.^39,40,41^ E6 also participates in multiple oncogenic events through protein–protein interactions. E6 interacts with Cylindromatosis (CYLD) deubiquitinase to inactivate the tumor suppressor CYLD and to activate the nuclear factor kappa-light-chain-enhancer of activated B cells (NF-κB) pathway in hypoxic conditions.^42^ E6 interacts with p300/cAMP response element binding protein (CREB)^43,44^ and interferon regulatory factor 3 (IRF-3)^45^ to regulate gene expression and with c-Myc to induce upregulation of human telomerase reverse transcriptase to promote cell immortalization.^46,47,48^ HPV16 E6 in the cytoplasm is also important for the oncogenic activity through its regulation of signal transduction by interactions with cytoplasmic E6BP (Erc55),^49^ E6TP1,^50,51^ tumor-necrosis factor (TNF) receptor 1^52^ and protein tyrosine phosphatase H1.^53^ In addition to its oncogenic activities, HPV16 E6 also protects HPV16-infected keratinocytes from the innate immune system by suppressing pro-IL-1β expression.^54^

The E7 oncoprotein consists of ∼100 aa. In HPV16 and 45, two CxxC zinc-binding motifs (Figure 1A) and a hydrophobic surface are essential for E7 homodimerization and protein stability.^55^ E7 is largely distributed in the nucleus, with a small fraction shuttling to the cytoplasm through two nuclear localization signals and a single nuclear export signal at the C-terminus.^56^ The N-terminus of E7 contains sequence similarity to a portion of the conserved region 1(CR1) region and the entire CR2 region of adenovirus E1A and the related sequences in SV40 T antigen. Oncogenic E7 binds pRB and the related pocket proteins p107 and p130 with high affinity via an LxCxE motif in CR2 (Figure 1A). Although low-risk or non-oncogenic E7 only weakly binds pRB, oncogenic E7 induces pRB degradation by interacting with the cullin 2 ubiquitin ligase complex.^57^ E7 induces aberrant cell cycle progression through upregulation of p21^58,59^ and p16.^60^ E7 also induces chromosomal instability and aneuploidy through association with γ-tubulin; this inhibits it from being recruited to the centrosome^61^ and complements the requirement for cyclin-dependent kinase 6 (CDK6), ERBB3, FYN, adaptor-associated protein kinase 1 and testis-specific serine kinase 2 for cell survival in colorectal cancer cells.^62^ Although E7 from both high-risk and low-risk HPVs interacts with p300,^63^ p300/CBP protein-associated factor (PCAF),^64^ steroid receptor co-activator 1^65^ and p600,^66^ these interactions are not sufficient for E7-mediated transformation.

### HPV oncogenes and RNA splicing

The HPV E6 and E7 oncogenes are juxtaposed in two different reading frames in the HPV genomes. Low-risk HPVs and high-risk HPVs utilize different strategies to express E6 and E7. In low-risk HPVs, such as HPV1, 2, 6 and 11, E6 and E7 are transcribed individually from two independent promoters. In contrast, E6 and E7 in high-risk HPVs, such as HPV16, 18, 31, 45 and 58, are transcribed as a single polycistronic E6E7 pre-mRNA from a single early gene promoter upstream of the E6 coding region (such as P_97_ in HPV16). Although early transcripts of both low-risk and high-risk HPVs contain an intron with alternative splice sites overlapping the E1 or E2 coding regions, and both utilize an early polyadenylation signal downstream of the E5 coding region for RNA polyadenylation, a striking feature of the high-risk HPV E6E7 polycistronic transcript is its unique E6 intron structure and alternative RNA splicing in the E6 coding region.^29^ The E6 transcript from low-risk HPVs does not have an E6 intron and thus does not undergo RNA splicing in the E6 coding region.^67^ In general, the E6 intron (also called intron 1) in high-risk E6E7 polycistronic pre-mRNAs features one major 5′ splice site (5′ ss) and one major 3′ splice site (3′ ss), and splicing of this intron disrupts the viral E6 ORF, preventing translation of full-length E6.^28,68^ Moreover, the E6 intron may extend into the E7, E2 or E4 coding regions through the use of an alternative 3′ ss further downstream of the E6 ORF. In the case of HPV16, alternative E6 intron splicing of the polycistronic E6E7 pre-mRNA leads to the production of seven RNA splicing isoforms, E6*I, E6*II, E6*III, E6*IV, E6*V, E6*VI and E6^∧^E7 in addition to a full-length, unspliced E6 mRNA (Figure 1B).^27,29^

As diagrammed in Figure 1B, the E6 intron of HPV16 bears three alternative 5′ ss in the E6 ORF and three alternative 3′ ss either in the E6 or E7 ORFs. The splicing of intron 1 from HPV16 E6E7 pre-mRNA is highly efficient and depends on intron definition. The majority of the spliced products in cervical cancer and its derived cell lines are E6*I, derived from splicing of the nucleotide (nt) 226 5′ ss to the nt 409 3′ ss. Preferential selection of this pair of splice sites over the other splice sites crossing over the intron minimizes the length of the intron in RNA splicing for energy saving^27,69,70^ and is dictated by an adenosine at nt 385 within the branch-point sequence AACAAAC in the virus genome.^27^ HPV16 E6*I encodes a truncated E6*I protein with 43 aa residues, which is in general expressed at levels below the detection threshold. Although the function of the HPV16 E6*I protein remains unknown, HPV18 E6*I appears to play a dominant negative role with regard to full-length E6 oncoprotein^71,72,73^ and to induce proteasomal degradation of PDZ proteins, including the tumor suppressor hDlg.^74^ Thus, E6*I splicing is not a viral strategy to produce a potent E6*I protein, but rather a strategy to create an E7 mRNA that can be translated into E7 oncoprotein.^28^ Because the E6 ORF is only two nucleotides upstream of the E7 ORF in the HPV16 genome, a scanning ribosome is unable to efficiently re-initiate E7 translation from an RNA containing the unspliced, full-length E6 ORF because translation termination of E6 and re-initiation of E7 translation has to occur within the two nucleotides. Subsequently, only the E6 oncoprotein is translatable from the unspliced, intact E6 ORF-containing RNA (Figure 1C).^27,68,75,76^ The same is true for translation of HPV18 E6 and E7. However, splicing that produces E6*I creates a premature stop codon immediately downstream of the splice junction and increases the distance between the E6*I ORF and the E7 ORF to >130 nts in both HPV16 and HPV18, a much better condition for scanning ribosomes to re-initiate E7 translation.^77^ Therefore, the most abundant spliced RNA, E6*I, actually functions as an E7 mRNA for efficient E7 translation (Figure 1C).^27,28,68^

In spite of the importance of E6 intron splicing in the production of E7, the regulatory mechanism underlying this splicing event remains largely unexplored. Epidermal growth factor activates extracellular regulated protein kinases 1/2 (Erk1/2), and this activation appears to suppress the HPV16 E6 intron splicing, resulting in increased E6, but decreased E7, translation.^76^ HPV16 E5 promotes epidermal growth factor receptor activation^78,79,80,81^ and therefore, may regulate splicing of the E6 intron through activation of the epidermal growth factor pathway. Both HPV16 E2 and E6 act as RNA-binding proteins and suppress splicing of the HPV16 E6 intron,^82^ suggesting the presence of a positive feedback loop of E6 production.

## MERKEL CELL POLYOMAVIRUS

### MCV and oncogenes

Polyomaviruses are distantly related to papillomaviruses and were formerly categorized with papillomaviruses into the papovavirus family, a taxonomy which is no longer used. Among the nine species of human polyomaviruses, MCV is the only one proven to be an etiologic agent of human cancer. MCV infection in immune-suppressed individuals can result in MCC, an aggressive form of nonmelanoma skin cancer. MCV has been identified in ∼80% of MCCs.^83^ Established MCC cell lines contain integrated MCV DNA encoding a mutant T antigen that prevents replication of the integrated virus from being autoactivated.^84^ This form of mutation in T antigen also affects epitope recognition by cytotoxic T cells.^85^ Although the other human polyomaviruses, BK polyomaviruse (BKV) and JC polyomaviruse (JCV) , KI polyomaviruse (KIV), WU polyomaviruse (WUV), H polyomaviruse 6 (HPyV6), H polyomaviruse 7 (HPyV7), H polyomaviruse 9 (HPyV9) and trichodysplasia spinulosa-associated polyomaviruses (TSV), are oncogenic in rodents and nonhuman primates, their association with human cancer remains unknown.

**Figure 2:**
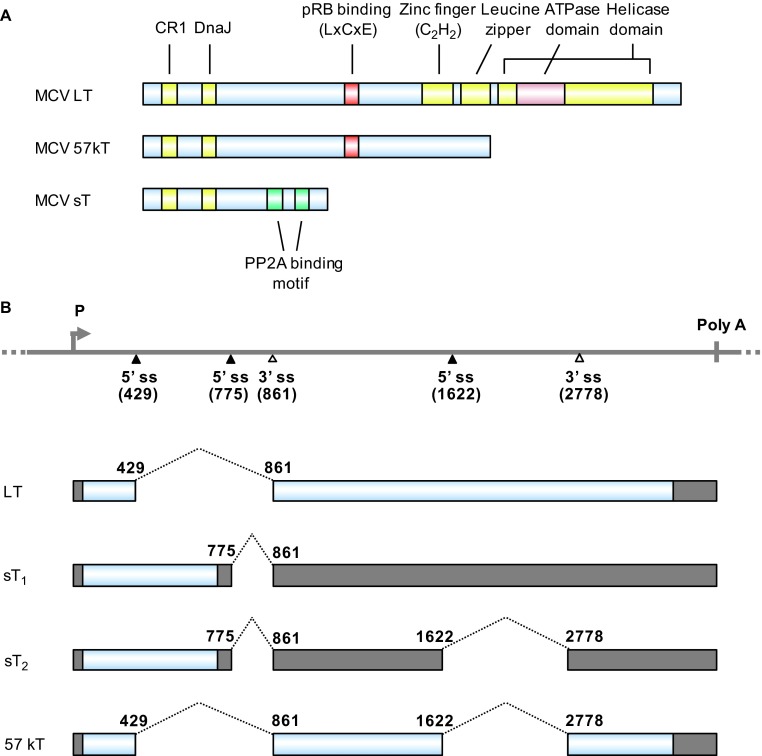
MCV T antigens and alternative RNA splicing. (**A**) Functional domains and motifs are indicated for the MCV large T antigen (LT), 57 kDa T antigen (57kT) and small T antigen (sT). (**B**) Scheme of alternative splicing of MCV T antigens. T antigen ORFs created by alternative RNA splicing (dashed lines) are in gray and 5′ and 3′ untranslated regions are in dark gray. Nucleotide positions are numbered according to the MCV genomic sequence (GenBank: NC_010277.1).

Polyomaviruses have been studied for their oncogenic activities since the discovery of SV40.^86^ SV40 is one of the most common latent viruses in rhesus monkeys and is capable of immortalizing and transforming rodent, but not human, cells. The oncogenicity of SV40 is mediated by large tumor (T) antigen (LT) and small T antigen (sT). ST is a spliced isoform of T antigens. The oncogenic activity of MCV, like that of SV40, is attributed to LT and sT. MCV LT and sT (Figure 2A) are well conserved with those of SV40. The MCV LT DnaJ domain binds to Hsc70 to promote proper folding of proteins after translation.^87^ MCV LT and 57kT antigens interact with pRB through their N-terminal LxCxE motif.^84^ The protein phosphatase 2A (PP2A) binding domain of MCV sT interacts with PP2A, as also seen with SV40 sT,^87,88^ but mutation of the PP2A binding domain does not impair the oncogenicity of MCV sT.^89^ MCV LT, but not SV40 LT, interacts with Vam6p.^90^ Vam6p binds to the N-terminus of MCV LT and changes its subcellular localization from the cytoplasm to the nucleus, but its association with the MCC tumorigenic process remains unclear.

### MCV oncogenes and RNA splicing

The early pre-mRNAs of MCV undergo alternative RNA splicing to produce four mRNA isoforms: LT, sT_1_, sT_2_ and 57kT (Figure 2B). To date, it remains unknown what RNA *cis*-element(s) or *trans-*acting cellular factors are involved in regulating the alternative RNA splicing. LT mRNA encodes LT antigen. Both sT_1_ and sT_2_ mRNAs encode sT, but differ in the length of the 3′ untranslated region. The 57kT mRNA corresponds to the 17kT mRNA in SV40 and encodes a 57-kDa protein missing a middle section of LT (Figure 2B). In contrast to SV40 sT and MCV LT, MCV sT exhibits oncogenic activity: it transforms rodent fibroblasts and promotes serum-independent growth of human fibroblasts.^89^ This is because MCV sT functions differently from SV40 sT; MCV sT promotes eukaryotic translation initiation factor 4E-binding protein 1 hyperphosphorylation and cap-dependent translation in a PP2A-independent manner,^89^ whereas SV40 sT induces dephosphorylation of 4E-binding protein 1 in a PP2A-dependent manner and inhibits cap-dependent translation.^91^ Moreover, MCV sT suppresses the activity of the E3 ubiqitin ligase SCF^Fbw7^, which degrades MCV LT, and stabilizes MCV LT through its LT-stabilization domain;^92^ SV40 sT does not have an LT-stabilization domain. Although inhibition of SCF^Fbw7^ stabilizes MCV LT, introduction of mutations in the sT LT-stabilization domain decreases LT protein levels and eliminates synergism in MCV DNA replication and sT-induced cell transformation.^92^ MCV sT is detected in 92% of MCC tissues, whereas MCV LT is only detected in 75% of the tissues.^87,89,93^ However, the full function of MCV sT in MCC cells requires other T antigens, such as LT and 57kT.^85,94^

## EPSTEIN-BARR VIRUS

### EBV and oncogenes

EBV is a human γ-herpesvirus best known as the cause of infectious mononucleosis,^95^ but it has been also associated with Burkitt lymphoma, nasopharyngeal carcinoma in southeastern Asia, natural killer (NK) cell leukemia, extranodal NK T-cell lymphoma and Hodgkin and non-Hodgkin lymphomas, accounting for ∼1% of cancer cases in humans worldwide.^21,96^ EBV is widespread in all human populations, with >90% of adults being serologically positive.^97^ A recent comprehensive analysis of the EBV genome revealed that EBV establishes a latent infection in nearly 100% of infected adults through numerous sites.^98^ In most cases, EBV infects B lymphocytes. Following EBV infection, B lymphocytes are transformed into lymphoblasts and become immortal. In rare cases, EBV also causes malignancy by infecting T cells and NK cells.^99,100,101^

Latent membrane protein-1 (LMP1) is considered to be a major viral oncogene of EBV. LMP1 is expressed in EBV-associated lymphoma and is essential for B-cell transformation and disruption of cellular signal transduction.^102,103,104^ Although EBV nuclear antigen 1 (EBNA1) is one of the earliest viral proteins expressed after infection and is the only latent protein consistently expressed in EBV-associated tumors, EBNA1 is not an oncoprotein.^105,106^ BamHI-A reading frame-1 is also an early gene, but it is expressed as a latent gene in most nasopharyngeal carcinomas.^107^ BamHI-A reading frame-1 may play an important role in nasopharyngeal oncogenesis, because it transforms rodent fibroblasts and primary epithelial cells and enhances tumor formation.^108,109,110,111^ The expression levels of EBV genes depend on the latency status of EBV, which is classified as latency 0, I, II or III. Hodgkin lymphomas are associated with latency II, in which EBNA1 and LMPs are expressed, but Burkitt lymphoma is associated with latency I, in which only EBNA1 is expressed. Nasopharyngeal carcinoma and T-cell and NK lymphomas display EBV latency I/II, an intermediate between latency I and II.

**Figure 3:**
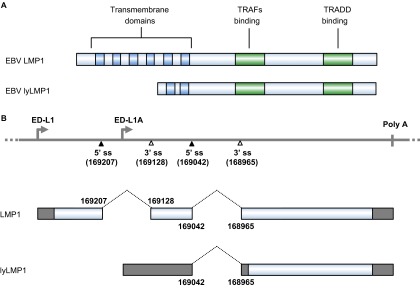
The EBV LMP1 oncoprotein and its lytic variant lyLMP1. (**A**) Functional domains and motifs are indicated for EBV LMP1 and lyLMP1 in the B95-8 EBV isolate. (**B**) EBV LMP1 ORF and lyLMP1 are produced by RNA splicing (dashed lines) from two separate transcripts derived either from the ED-L1 promoter or the ED-L1A promoter. Nucleotide positions are numbered according to the genomic DNA sequence of the B95-8 EBV isolate (GenBank: V01555.2). TRAF, TNF receptor-associated factor; TRADD, tumor necrosis factor receptor associated death domain protein.

LMP1 is a 62-kDa integral membrane protein and contains 386 aa residues, with a short cytoplasmic N-terminus of 24 aa, six transmembrane domains of 162 aa (from aa 25 to aa 186) and a cytoplasmic C-terminus of 200 aa (Figure 3A).^112,113^ LMP1 immortalizes and transforms human B cells, but its oncogenic activity can be interfered by lytic LMP1 (lyLMP1), a truncated form of LMP1 with 258 aa residues expressed in the lytic phase.^114^ LMP1 drives proliferation of EBV-infected B cells by signaling within the B cells without any ligand for LMP1. The pathway downstream of LMP1 overlaps with that of the CD40 receptor,^115^ which delivers signaling through TNF receptor associated factors and Janus kinase 3 to activate NF-κB, AP-1, STAT-1, CD83 and CD95. In clonal populations, LMP1 levels vary among cells by more than 100-fold. When expressed at an intermediate level, LMP1 signals through NF-κB to promote cell proliferation, but when expressed at a high level, LMP1 inhibits protein synthesis by activating double-stranded RNA-activated protein kinase-like endoplasmic reticulum kinase (PERK) to induce eukaryotic initiation factor 2α phosphorylation followed by activating transcription factor 4 (ATF4) upregulation. ATF4, in turn, activates the LMP1 promoter.^104^

Although the transmembrane domains of LMP1 activate B-cell apoptosis, the carboxy-terminal domain of LMP1 blocks this effect.^116^ In general, LMP1 activation leads to overexpression of antiapoptotic molecules, such as B-cell CLL/lymphoma 2 (Bcl-2),^117^ myeloid cell leukemia 1 (Mcl-1)^118^ and BCL-2-related protein A1 (Bcl2A1/Bfl-1),^116,119^ and blocks p53-mediated apoptosis through the induction of anti-apoptotic protein A20,^120^ unfolded protein response (UPR)-induced apoptosis in B cells^116^ and ubiquitin C-terminal hydrolase L1 (UCH-L1) with oncogenic properties.^121^ Complementary to its proliferative function, LMP1 inhibits proapoptotic factors such as Bax^122^ and induces autocrine factors, such as chemokine (C–C motif) ligand 3 (CCL3) and chemokine (C–C motif) ligand 4 (CCL4), to promote cell proliferation.^123^ LMP1 also enhances cancer cell motility by upregulating TNF α-induced protein 2 (TNFAIP2) through NF-κB activation.^124^ Moreover, LMP1 regulates miRNAs to exert both positive and negative roles on cell proliferation. LMP1 upregulates miR-29b to suppress TCL1 oncogene expression,^125^ but downregulates miR-203 to increase E2F transcription factor 3 (E2F3) and cyclin G1 expression^126^ and miR-15a to promote MYB and cyclin D1 expression.^127^

### EBV LMP1 and RNA splicing

The LMP1 gene is transcribed from either the ED-L1 or ED-L1A promoter within the BamHI-N region of the EBV genome^112,128^ and produces two LMP1 isoforms from the two alternative promoters (Figure 3B). Double splicing of ED-L1 pre-mRNA produces the LMP1 ORF encoding 386 aa residues, whereas single splicing of ED-L1A pre-mRNA produces the lyLMP1 ORF encoding 258 aa residues starting from the 129th methionine of LMP1 in B95-8 EBV isolates.^129,130^ Thus, lyLMP1 differs from LMP1 by lacking the N-terminal 129 aa residues of LMP1. However, the lyLMP1 transcript is non-coding in most EBV isolates^131^ and is upregulated during the lytic phase of EBV replication following activation of the lytic promoter ED-L1A.^132,133^ In contrast to LMP1, lyLMP1 has no transforming activity in rodent cells, nor does it alter the phenotypes of human B lymphocytes.^134^ The biological activity of lyLMP1 appears to be the negative regulation of LMP1-signaling pathways and LMP1-mediated oncogenesis.^114,135^

What regulates splicing of LMP1 RNA remains largely unexplored. EBV EB2 (also called SM) functions as a *trans-*acting factor^136,137^ in regulating RNA splicing via its interaction with the cellular oncogenic splicing factor SRSF3 (serine/arginine-rich splicing factor 3).^138^ Although EBV EB2 is not expressed in the latent stage of EBV infection, increased SRSF3 expression in lymphoma might play a role in LMP1 splicing and is worth investigating.

## HUMAN T-CELL LEUKEMIA VIRUS 1

### HTLV-1 and oncogenes

HTLV-1 is a delta retrovirus and is associated with ATL. Both HTLV-1 and ATL are endemic within the southwestern part of Japan, the Caribbean basin, and South Africa. Approximately 1%–5% of individuals with HTLV-1 infection develop ATL after 20–30 years of latency.^139,140^ Discovery of HTLV-1 occurred independently in the United States and Japan in the late 1970s. A retrovirus with type-C morphology was isolated from the blood of an African-American patient with cutaneous T-cell lymphoma and named human cutaneous T-cell lymphoma virus.^13^ In parallel, adult T-cell leukemia was found in the southwestern part of Japan,^141,142^ and a retrovirus with type-C morphology was also isolated from T cells from those patients and named adult T-cell leukemia virus.^14,15,143,144^ The two viruses were later found to be identical, and are now referred to as HTLV-1. Three related viruses, HTLV-2, -3 and -4, have also been reported. HTLV-2, which was isolated from a hairy T-cell leukemia, immortalizes human T cells *in vitro*,^145^ although its association with human disease has not yet been established. HTLV-3 and -4 were detected from their genome sequences, but their relation to human disease also remains unknown.^146,147^

**Figure 4:**
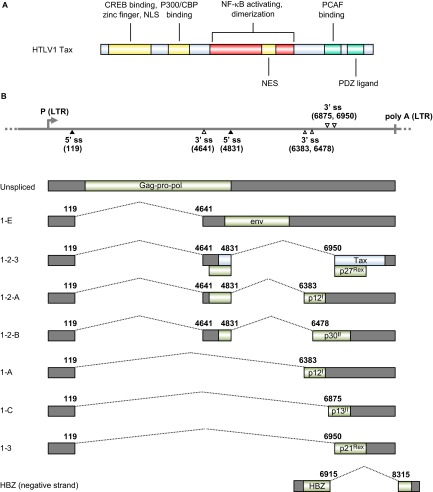
HTLV-1 viral RNA splicing and Tax oncogene expression. (**A**) Functional domains and motifs are indicated for the HTLV-1 Tax oncoprotein. (**B**) Alternative RNA splicing products of HTLV-1 viral genes. Seven positive-strand transcripts (1-E, 1-2-3, 1-2-A, 1-A, 1-2-B, 1-C and 1-3) and one negative strand transcript (HBZ) are produced by alternative RNA splicing of the transcripts derived from either a 5′ or 3′ long terminal repeat (LTR). The HTLV-1 Tax ORF in blue is present in the 1–2–3 mRNA, which is spliced from the nt 119 5′ ss to the nt 4641 3′ ss and then from the nt 4831 5′ ss to the nt 6950 3′ ss. Nucleotide positions are numbered according to the HTLV-1 genomic RNA sequence, starting from the first nucleotide in the pre-mRNA.^153^

*Tax*, a viral oncogene of HTLV-1, encodes a 40-kDa nuclear phosphoprotein consisting of 353 aa residues and confers the transforming properties of HTLV-1. Tax immortalizes T lymphocytes and induces leukemia in transgenic mice.^148,149^ It promotes viral transcription from a promoter located within the long terminal repeat (LTR).^150,151,152^ The N-terminus of Tax (Figure 4A) directly binds to CREB to form a ternary complex of TRE–Tax–CREB (Tax-responsive element–Tax–CREB) within the viral promoter.^154,155^ Binding of Tax to CREB enhances CREB homodimerization, which strengthens its association with promoter DNA.^156^ TRE activation requires Tax homodimerization, which involves CREB homodimerization. Tax also recruits the transcriptional co-activators CREB-binding protein (CBP) and p300^157,158,159^ and PCAF.^160,161^ Tax transactivates specific cellular transcripts by interacting with DNA-binding serum responsive factor, which recruits Tax to specific cellular promoters such as those of c-FOS, EGR-1 and EGR-2. ^162,163,164^ Tax activates the NF-κB pathway^165^ to enhance the expression of IL-2,^166,167^ IL-2R,^168^ IL-15^169^ and IL-15R,^170^ leading to the formation of an autocrine loop for the proliferation of HTLV-1-infected T cells. Tax inactivates the tumor suppressor p53^171^ and its homologues p73α and β,^172,173^ but activates canonical Wnt signaling in the presence of a Wnt pathway-associated protein, disheveled-associating protein with a high frequency of leucine residues (DAPLE).^174^ Tax binds INT6/EIF3E, a subunit of the translation initiation factor eukaryotic initiation factor 3, and upstream frameshift protein 1 (UPF1) to inhibit nonsense-mediated mRNA decay.^175^

Despite its oncogenic activity, Tax expression is low or undetectable in tumor cells from adult T-cell leukemia.^176,177,178^ The current consensus is that Tax is necessary for initiating cell transformation, but in later stages, acquired genetic and epigenetic changes and alternative growth-promoting pathways replace the roles of Tax to maintain adult T-cell leukemia when Tax is no longer expressed.

### HTLV-1 RNA splicing and production of tax mRNA

Reverse transcription of HTLV-1 genomic RNA into DNA and subsequent DNA integration into the host cell genome is a necessary step for retroviral replication. All HTLV-1 genes, except the newly described HTLV-1 basic leucine zipper factor (HBZ) gene, are transcribed from a single promoter within the 5′ LTR as a single polycistronic pre-mRNA transcript that is polyadenylated by using a polyadenylation signal in the 3′ LTR.^67^ HBZ is encoded by an antisense transcript derived from a minor promoter in the 3' LTR of the HTLV-1 genome.^179^ Both the major polycistronic transcript and the minor monocistronic antisense transcript contain introns and are subject to regulation by RNA splicing.^180^ The major polycistronic pre-mRNA transcript covers almost the entire viral genome. Gag-Pro-Pol, a precursor polyprotein, is translated from the unspliced form of the polycistronic RNA. However, the RNA also has two introns and three exons with two alternative 5′ ss at nt 119 and nt 4831, and five alternative 3′ ss at nts 4641, 6383, 6478, 6875 and 6950. The HBZ monocistronic pre-mRNA has only one intron, and the antisense HBZ transcript undergoes RNA splicing from nt 8315 to 6915.^179^ This leads to extensive alternative RNA splicing and produce nine types of spliced and unspliced mRNAs (Figure 4B). The Tax ORF is produced by double RNA splicing of the polycistronic RNA, with the first intron splice from nt 119 to 4641 and the second intron splice from nt 4831 to 6950 in the spliced 1–2–3 form of the RNA (Figure 4B). What controls or regulates the alternative RNA splicing in HTLV-1 infection and gene expression remains to be investigated. RNA *cis*-elements and *trans*-acting factors responsible for HTLV-1 alternative RNA splicing remain unknown.

## KAPOSI'S SARCOMA-ASSOCIATED HERPESVIRUS

### KSHV and viral genes with oncogenic activities

KSHV or human herpesvirus 8 was discovered in 1994 as a member of the human gamma herpesvirus family, joining EBV.^20,181^ Infection of immune-compromised individuals with KSHV has been associated with the development of endothelial cell-derived Kaposi's sarcoma and at least two B cell lymphoproliferative diseases: primary effusion lymphoma and multicentric Castleman's disease.^21^ However, studying KSHV pathogenesis and oncogenesis has been hindered by lack of a meaningful animal model and susceptible cell culture, although primary endothelial cells provide limited KSHV replication ^182,183^. Two immortalized cell lines, KS Y-1 and SLK, were once used for KS and KSHV studies,^184,185^ but the KS Y-1 cell line is cross-contaminated with the T24 urinary bladder cancer cell line (ATCC HTB-4) and SLK is a contaminant of a known renal carcinoma cell line, Caki-1.^186^ Thus, neither are of endothelial origin. Primary rat embryonic metanephric mesenchymal precursor cells are susceptible to KSHV infection and transformation, but only shed a limited number of infectious virions.^187^ Primary effusion lymphoma-derived B-cell lines are commonly infected with KSHV at the latent stage and can be induced to produce low levels of KSHV virions,^188,189^ but primary B lymphocytes from peripheral blood or tonsillar tissue are refractory to infection by KSHV,^190,191^ and their limited infection may require cocultivation with KSHV-positive cells.^185^ The human MC116 cell line, which has characteristics of transitional B cells,^192^ can be infected with limitation by the KSHV.219 virus, which also infects Vero cells and produces high yields of virus.^193^ Humanized bone marrow, liver and thymus (BLT) mice infected by inoculation with KSHV.219 virus via the oral and vaginal routes could be a useful model for studying the pathogenesis and transmission of KSHV.^194^

KSHV encodes several important proteins that have some oncogenic activity for inducing cell proliferation, immortalization, transformation and signaling; cytokine production; immune evasion; antiapoptosis activity; and angiogenesis. These include the viral latent proteins latent-associated nuclear antigen (LANA), vFLIP (a FADD (Fas-associated protein with death domain)-like interferon converting enzyme or caspase 8 (FLICE) inhibitory protein), and vCyclin and the viral lytic proteins G-protein coupled receptor (vGPCR) interferon regulatory factor 1 (vIRF-1) and K1. Although the true oncogenic nature of each protein remains to be defined, accumulating evidence indicates that each of them contributes some aspect to KSHV oncogenesis. Thus, a full spectrum of KSHV-induced malignancy might require multiple oncogenic products to work together in the presence of host and environmental cofactors. For example, both LANA and vIRF-1 target the cellular tumor suppressor p53.^195,196^ LANA also inhibits pRB and PP2A.^197^ vCyclin, an activator of CDK4/6,^198^ downregulates p27^kip1^, a CDK inhibitor,^199^ and counters the senescence/G1 arrest response that results from NF-κB hyperactivation.^200^ Both vFLIP and K1 activate the NF-κB signal pathway to prevent B cell apoptosis.^201,202^ vGPCR and K1 affect the AKT and NF-κB signal pathways^203,204,205^ and contribute to angioproliferative and inflammatory Kaposi's sarcoma lesions.^206,207^ More importantly, the latent locus of the KSHV genome by itself shows B cell oncogenicity in transgenic mice.^208^

### Transcription and RNA splicing in the KSHV latent locus

**Figure 5:**
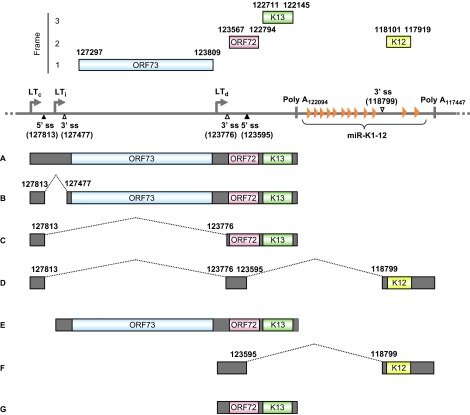
Transcription map of KSHV ORF73 (LANA), ORF72 (v-Cyclin), ORF71 (K13, v-FLIP) and K12 (kaposin). The diagram shows major alternative splicing products of KSHV ORF73, ORF72, ORF71 and K12. The heavy line indicates the corresponding latent locus region of KSHV genome. Three promoters (LT_c_, constitutive promoter; LT_i_, RTA-inducible promoter; LT_d_, downstream promoter), two polyadenylation signals (poly A at nt 122094 and nt 117432) and alternative 5′ ss and 3′ ss in this region are indicated. Twelve viral miRNAs (miR-K1-12) clustering downstream of the K13 ORF are shown as orange triangles. Boxes above the line represent ORFs for ORF73, ORF72, K13 and K12. Below the line are common RNA species (**A**–**G**) derived from alternative RNA splicing from this region. Nucleotide positions are indicated according to KSHV genome (GenBank: U75698.1).^181^

The KSHV latent locus encodes multiple viral latent genes, including LANA (ORF73), vCyclin (ORF72), vFLIP (ORF71 or K13), all viral miRNAs and kaposin (K12). Expression of these latent genes from this locus is driven by three promoters (Figure 5): two constitutive latent promoters (LT_c_ and LT_d_) and an inducible latent promoter (LT_i_) that is inactive but can be induced by the lytic switch protein RTA encoded by ORF 50.^209^ Although LT_d_ is constitutively active, mirroring LT_c_, its transcriptional activity can be further boosted by expression of RTA, reminiscent of LTi.^209^ As diagrammed in Figure 5, both the LT_c_ and LT_i_ transcripts are tricistronic and are polyadenylated by using a common poly A (pA) signal at nt 122094 in the virus genome. The LT_c_ transcript has an intron with two alternative 3′ ss and is thus subject to regulation by alternative RNA splicing, whereas the LT_i_ transcript does not have an intron and is not spliced. Consequently, LANA is expressed from the unspliced RNA species A or E or the spliced RNA species B by using a proximal 3′ ss that preserves the intact ORF73 ORF. In KSHV-infected cells, the majority of LANA is translated from abundant A RNA. Viral vCyclin and vFLIP are expressed mainly from spliced C RNA derived by selection of a distal 3′ ss for RNA splicing, with vFLIP being expressed from this transcript by using an internal ribosome entry site residing in the vCylcin ORF.^210^ Occasionally, an LT_c_ transcript is double spliced to the K12 ORF region by using another pA signal further downstream at nt 117432 (RNA species D).^211^ This last splicing strategy presumably enables the LT_c_ transcript to encode K12. The LT_d_ transcript can be spliced to the K12 ORF for polyadenylation to encode K12, or it can remain unspliced, in which case it is polyadenylated by using the same pA signal as the LT_i_ RNA E to encode vCyclin and vFLIP. The intron from the LT_d_ RNA species F could be a source of KSHV miRNAs.^209,212^ However, very little is known about the splicing regulation of these latent transcripts.

## HEPATITIS B VIRUS AND HEPATITIS C VIRUS 

### HBV and hepatocellular carcinoma

More than 50% of liver cancers worldwide are attributed to HBV infection. HBV is a DNA virus whose ∼3.2 kb, partially double-stranded, circular DNA genome is covered by a nucleocapsid core and an outer lipid envelope. The HBV envelope consists of the small (S), middle (M) and large (L) envelope proteins, which are multiple-transmembrane proteins that share the same C-terminal domain (corresponding to the S protein) but which differ at their N-terminal domains. The HBV genome encodes four viral genes: C (HBcAg), X (HBx), P (DNA polymerase) and S (HBsAg). The S gene encodes a long ORF with three in-frame ‘start' (ATG) codons that divide the gene into three sections, pre-S1, pre-S2 and S. HBV enters susceptible liver cells when the receptor-binding region of pre-S1 specifically interacts with the functional cellular receptor NTCP (sodium taurocholate cotransporting polypeptide), a multiple transmembrane transporter predominantly expressed in the liver; this results in liver infection and virus replication.^213^ Persistence of chronic HBV infection for decades is linked to liver cirrhosis and the development of hepatocellular carcinoma (HCC). Although HBV infection is prevalent worldwide, chronic HBV infection is most common in East Asia through perinatal transmission and in Africa via childhood infection.^214,215,216^ Continuous cycling of immune clearance and regeneration of hepatocytes during chronic HBV infection is considered a risk factor for HCC development. HBx is a transactivating protein that interacts with protein arginine methyltransferase 1 to regulate the expression of cellular genes,^217^ stimulating cell growth-promoting genes and inactivating growth regulating molecules. HBx regulates cell cycle progression and cell proliferation by activating NF-κB;^218^ upregulating vascular endothelial growth factor receptor 3 in hepatocarcinogenesis;^219^ stimulating cell migration, growth in soft agar, and spheroid formation; and promoting ‘stemness' in the pathogenesis of HCC.^220^ HBx and aflatoxin B1 synergistically cause hepatitis, steatosis, and liver hyperplasia in transgenic zebrafish.^221^

### HBV RNA splicing and HCC

**Figure 6:**
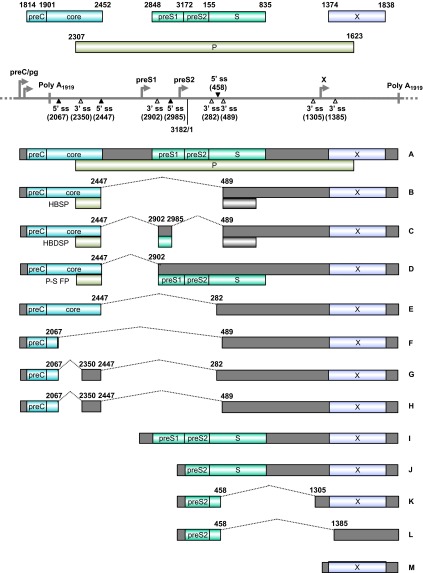
Genome structure and transcription map of HBV. The full-length, circular HBV genome (GenBank: X02496.1)^222^ is illustrated in a linear form for better presentation of tail-to-head (3182/1) junction, four promoters (preC/pg, preS1, preS2 and X), a single poly A site at nt 1919, four alternative 5′ ss (filled triangles) and six alternative 3′ ss (open triangles). Above the linear HBV genome are viral ORFs, each with the numbered positions of the first nt of the start codon (including the in-frame start codon) and the last nt of the stop codon. Below the linear HBV genome are the RNA species (**A**–**M**) commonly derived from alternative RNA splicing, with the coding exons in colored boxes and the non-coding exons in grey boxes. The dotted lines indicate the introns and splicing directions for each RNA species, with the mapped splice site positions being numbered by nt positions in the HBV genome. HBSP, HBDSP and P-S FP are the ORFs created by alternative splicing of the preC/pg RNA. HBSP, hepatitis B splice-generated protein; HBDSP, hepatitis B doubly spliced protein; P-S FP, polymerase-surface fusion protein.

Transcription of HBV RNAs is controlled by four different promoters: preCore/pre-genomic (preC/pg, for the 3.5-kb core RNA that codes for viral core protein and DNA polymerase), pre-S1 (for the 2.4-kb pre-S1 RNA that encodes pre-S1), major pre-S2/S (for the 2.1-kb pre-S2/S RNAs that encode the pre-S2 and S proteins) and X (for the 0.7-kb RNA encoding HBx antigen) (Figure 6).^223,224^ However, all HBV RNA transcripts are polyadenylated by using a single pA signal downstream of the HBx termination codon. With such a genome structure, HBV encodes specific sequence elements to promote extensive splicing of HBV pre-genomic/preC (pg/preC), pre-S1 and pre-S2/S RNAs, a common event during chronic infection, leading to the production of seven additional common splicing variants of pg/preC RNAs and two additional splicing variants of pre-S2/S RNAs (Figure 6).^225,226^ A 2.2-kb singly spliced (spliced from nt 2447–489, Figure 6 RNA B) or doubly spliced (spliced from nt 2447 to nt 2902 and then from nt 2985 to nt 489, Figure 6 RNA C) HBV pg/preC RNA is most common in cultured cells. It appears that this single or double splicing event is regulated by two RNA *cis*-elements, the post-transcriptional regulatory element at nts 1217–1582^225,227^ and the intronic splicing silencer (ISS) at nts 2591–3163,^228^ and by PTB-associated splicing factor in interaction with the post-transcriptional regulatory element.^229^ The singly spliced RNA encodes an hepatitis B splice-generated protein (HBSP), which contains a small portion of the N-terminal viral polymerase fused with a new ORF produced by RNA splicing.^230^ HBSP is associated with pathogenesis after HBV infection and increases the risk of development of HCC by promoting viral replication and protein production^231^ and liver fibrosis.^232^ HBSP expression activates the HBV S1 promoter, S2 promoter, enhancer I and core upstream regulatory sequences.^233^ HBSP interacts with cathepsin B to enhance hepatoma cell migration and invasion^234^ and induces T-cell responses in human leukocyte antigen-transgenic mice and HBV-infected patients.^235^ The doubly spliced RNA encodes a ∼15-kDa hepatitis B doubly spliced protein which may play a role in viral transcription and DNA replication.^233,236^ Polymerase-surface fusion protein (P-S FP), which is encoded by another spliced transcript derived by splicing from nt 2474 to 2902 of pg/preC RNA (Figure 6, RNA D), might regulate HBV replication.^237,238^ The functions of the spliced pre-S2/S RNAs remain unknown, and the HBx transcript does not undergo RNA splicing.

### HCV and oncogenesis

HCV is a positive-strand RNA virus with a ∼9.6-kb genome. The HCV genome encodes a single ORF for a polyprotein of about 3000 aa residues, which is cleaved into 10 different viral proteins. In order from the N-terminus to C-terminus, these are a viral core protein, the two envelope proteins E1 and E2, and seven non-structural proteins p7 (NS1)–NS2–NS3 (protease/RNA helicase)–NS4A–NS4B–NS5A–NS5B (RNA polymerase). The HCV lifecycle is restricted to the cytoplasm and is not regulated by nuclear RNA splicing. Accumulation of HCV RNA in the infected cells requires an interaction of host miR-122 with the 5′-non-coding region of HCV RNA.^239,240^ HCV grows in Huh-7-derived cell lines.^241,242,243^ Similar to HBV, chronic HCV infection over decades is strongly associated with liver cirrhosis and HCC, and chronic inflammation and regeneration of hepatocytes through the immune response are prognostic risk factors. At least four of the HCV gene products, the viral core, NS3, NS4B and NS5A, exhibit transformation potential in tissue culture, and several oncogenic pathways can be altered by the expression of these HCV proteins, thus presumably contributing to HCC development.^244,245,246^ Today, approximately ∼94% of chronic HCV genotype 1 infection in adults can be cured in 8 weeks by a once-daily fixed-dose combination of the NS5A inhibitor ledipasvir 90 mg and the nucleotide analog polymerase (NS5B) inhibitor sofosbuvir 400 mg.^247,248,249^

## REMARKS

The discoveries in the past two decades of KSHV and MCV, the introduction of HPV vaccines for the prevention of cervical cancer and the successful growth of HCV in Huh-7-derived cell lines and treatment of HCV chronic infection with sofosbuvir/ledipasvir are historical landmarks in tumor virology and human cancer research. These remarkable successes reiterate that the global fight against human cancers will continue to receive great support from our tremendous efforts in searching for new tumor-causing viruses and in understanding the basic biology of tumor viruses. Oncogenes, signal transduction and RNA splicing were all discovered by tumor virologists in the late 1970s and early 1980s and have had tremendous impact on today's cancer research portfolio around the world. As discussed in this review, all tumor viruses express oncogenes (HPV, EBV, MCV and HTLV-1) or genes with oncogenic activities (KSHV, HBV and HCV) that immortalize and transform host cells. However, expression of these defined oncogenes, although regulated at the transcriptional level, is also profoundly under the control of alternative RNA splicing at the post-transcriptional level, and to date we know only a little about the mechanisms that regulate RNA splicing in the context of viral infection and viral oncogenesis of host cells. Exploring RNA *cis-*elements and cellular *trans-*acting splicing factors in the regulation of alternative RNA splicing of tumor viruses has shed some light on the mechanisms over the past 18 years and will most likely continue to be a prospective focus in viral oncogene research. Gaining further understanding of how the cell- or tissue-specific expression of splicing factors is involved in splicing of viral oncogene RNA will be a formidable challenge, but will provide some fundamental insight into how alternative RNA splicing and viral gene expression take place in a cell- or tissue-specific manner. Looking back on 100 years of tumor virology history, we have learned a great deal about human carcinogenesis from many landmark discoveries in tumor virology. In looking forward into the twenty-first century, understanding and manipulation of RNA splicing in the development and control of human cancers will definitely trigger another wave of discoveries in RNA biology. Human tumor viruses will inevitably be windows into this complex nature.

## Figures and Tables

**Table 1:** Oncogenic human viruses and viral oncogenes.

Tumor virus	Associated cancer(s)	Viral oncogenes or potential oncogenes*
High-risk HPVs	Cervical cancer, anal cancer, penile cancer, vaginal cancer, oropharyngeal cancer	E6, E7
MCV	Merkel cell carcinoma	T antigens
HTLV-1	Adult T-cell lymphoma	Tax
EBV	Burkitt lymphoma, Hodgkin lymphoma, non-Hodgkin lymphoma nasopharyngeal cancer, T-cell and NK lymphoma	LMP1
KSHV	Kaposi's sarcoma, primary effusion lymphoma, multicentric Castleman's disease	LANA, vFLIP, vCyclin,vGPCR, vIRF-1, K1
HBV	Hepatocellular carcinoma	HBx
HCV	Hepatocellular carcinoma	Core protein, NS3, NS4B, NS5A

* For KSHV, HBV and HCV, potential viral oncogenes are presented.
